# A Europium Nanosphere-Based Time-Resolved Fluorescent Immunochromatographic Assay for the Rapid Screening of 4,4′-Dinitrocarbanilide: Aiming at Improving Strip Method Performance

**DOI:** 10.3390/bios13050518

**Published:** 2023-05-04

**Authors:** Ming Zou, Yongkang Yin, Liuchuan Guo, Qidi Zhang, Jinyan Li, Hong Zhang, Qian Song, Zhaojie Li, Li Wang, Xiang Ao, Xiao Liang

**Affiliations:** 1 College of Veterinary Medicine, Qingdao Agricultural University, No. 700 Changcheng Road, Qingdao 266109, China; 2 College of Food Science and Engineering, Qingdao Agricultural University, No. 700 Changcheng Road, Qingdao 266109, China; 3 Basic Medical College, Qingdao University, No. 308 Ningxia Road, Qingdao 266071, China

**Keywords:** NIC, DNC, residue, TRFICA, chicken muscle

## Abstract

Considering that the strip method is simple and convenient for users, a Europium nanosphere-based time-resolved fluorescent immunochromatographic assay (TRFICA) for the rapid screening of 4,4′-dinitrocarbanilide (DNC) was developed to improve the performance of strip assays. After optimization, TRFICA showed IC_50_, the limit of detection, and cut-off values of 0.4, 0.07, and 5.0 ng mL^−1^, respectively. No significant cross-reactivity (CR < 0.1%) with 15 DNC analogs was observed in the developed method. TRFICA was validated for DNC detection in spiked chicken homogenates, and recoveries ranged from 77.3% to 92.7%, with coefficients of variation of <14.9%. Moreover, the time needed for the detection procedure, including the sample pre-treatment, was less than 30 min for TRFICA, which had never been achieved before in other immunoassays. The newly developed strip test is a rapid, sensitive, quantitative, and cost-effective on-site screening technique for DNC analysis in chicken muscle.

## 1. Introduction

Coccidiosis is a widespread and economically significant livestock disease caused by protozoan parasites of the genus Eimeria [1]. Minor infections cause poor feed conversion and weight gain, whereas major infections can cause significant mortality [2]. Therefore, in addition to the diagnosis and treatment of animal diseases, strict biological prevention and control from the source are also necessary. Feed contaminated with mycotoxins may cause the release of toxic substances in edible tissues such as meat, fish, and dairy products [3], seriously endangering human health and safety. Aflatoxin B1 (AFB1), the most toxic secondary metabolite produced by Aspergillus flavus, has become a major food safety issue worldwide due to its contamination of poultry feed [4]. In terms of biological prevention and control, the assessment of residual toxicity of multiple veterinary drugs in animal-derived foods based on endocrine disruptors using a high-throughput exposure (HTE) model [5] and a risk assessment of low-dose dietary-related exposures concludes that a tolerable daily intake (TDI) is unlikely to have genotoxic effects leading to carcinogenicity [6], to achieve the goal of ensuring human food safety and life health safety. Nicarbazin (NIC), an equimolecular mixture of 4,4-dinitrocarbanilide (DNC) and 2-hydroxy-4,6-dimethylpyrimidine, is a synthetic coccidiostat used globally to cure coccidiosis in animals, especially poultry. The widespread use of NIC causes its residues to be present in foods of animal origin, such as broiler tissues. Its residues can seriously affect the health of poultry and humans, such as by causing adverse heat stress and death in broilers and chronic toxicity in humans. Therefore, DNC is considered the residue of concern in edible chicken tissues [7]. The maximum residue limits (MRLs) for DNC in food matrices were established to assure food safety in poultry production. The Joint FAO/WHO Expert Committee on Food Additives in New Zealand and China has recommended an MRL of 200 μg of DNC/kg in all broiler tissues, and the EU recommended an MRL of 4000 μg of DNC/kg. Therefore, it is imperative to develop low-cost, sensitive, and effective methods for the detection of DNC in animal-derived foods. Multiple technologies have been applied to detect residues of DNC in food matrices. Instrumental methods, such as high-performance liquid chromatography (HPLC) [8,9,10], high-performance liquid chromatography-tandem mass spectrometry (HPLC-MS/MS) [2,11,12,13], and ultraperformance liquid chromatography-tandem mass spectrometry (UPLC-MS/MS) [14] are accurate and specific, but their experiments are complex and not suitable for on-site screening. Immunoassay, such as enzyme-linked immunosorbent assay (ELISA) [15,16,17], has the advantages of high throughput screening but has significant limitations and is not suitable for on-site screening. Compared with the other methods, the immunochromatographic assay (ICA) is a more portable and faster assay [18]. Different types of fluorescent nanobeads, such as quantum dots [19], fluorescent microspheres [20], and up-conversion phosphors [21], among them, time-resolved fluorescent nanoparticles have excellent fluorescence properties. Lanthanide labels possess long decay lifetimes, can be dissociated by altering the solution pH, resulting in a shift to a new highly fluorescent chelate, and have a large Stokes shift, high quantum yields, sharp emission profiles, and narrow emission peaks. Although the quantum yields are lower with the use of organic fluorescence dyes, packing them into nanosphere capsules can increase fluorescence thousands of times [22,23]. Therefore, these attributes enable time-resolved fluorescence immunochromatography (TRFICA) labeled with fluorescent microspheres to have higher sensitivity, lower matrix interference, and better reproducibility and stability.

As shown in previous studies, TRFICA has been successfully applied to the quantitative detection of contaminants in food. Shen et al. developed a TRFICA for detecting chlorpromazine residues in pork, with a limit of detection (LOD) of 0.32 μg kg^−1^ and a wide dynamic range of 0.46–10.0 μg kg^−1^ [24]. Du et al. used two fluorescent labels to establish a dual-labeled TRFICA for analyzing diethyl phthalate and dibutyl phthalate in aquatic environments. The LODs of the assay were 4.9 ng mL^−1^ and 3.9 ng mL^−1^, respectively [25]. A TRFICA that uses lanthanides as labels, such as Eu nanospheres (EuNPs), is one of the most promising immunoassay methods. Xu et al. developed a TRFICA for the rapid quantification of FB1 in different grains, with a LOD of 8.26 μg kg^−1^ and a wide detection range of 13.81–1000 μg kg^−1^ [26]. Ma et al. established a TRFICA method for determining the contaminant in milk samples, with a LOD and limit of the quantity of 3.05 ng mL^−1^ and 6.63 ng mL^−1^, respectively [27]. Therefore, this technology is widely used in the detection of specific pollutants with the advantages of high cost-effectiveness, high selectivity, high sensitivity, simple operation, and a wide dynamic range.

In the current study, a TRFICA with high sensitivity and low cost for screening DNC was innovatively developed. The reaction conditions for Eu-NP-mAb probe conjugation, coating antigen, recombinant buffer, coating buffer, incubation time, sample dilution buffer, and immunochromatographic bands were optimized to improve the reaction performance. Under optimal experimental conditions, the standard curve of the DNC was developed. TRFICA had high specificity for DNC and neglected cross-reactivity in drugs with 15 similar structures. TRFICA could be used in chicken detection without complicated sample preparation, and the time needed for the detection procedure, including sample pre-treatment, was less than 30 min.

## 2. Materials and Methods

### 2.1. Reagents and Materials

**Figure 1:**
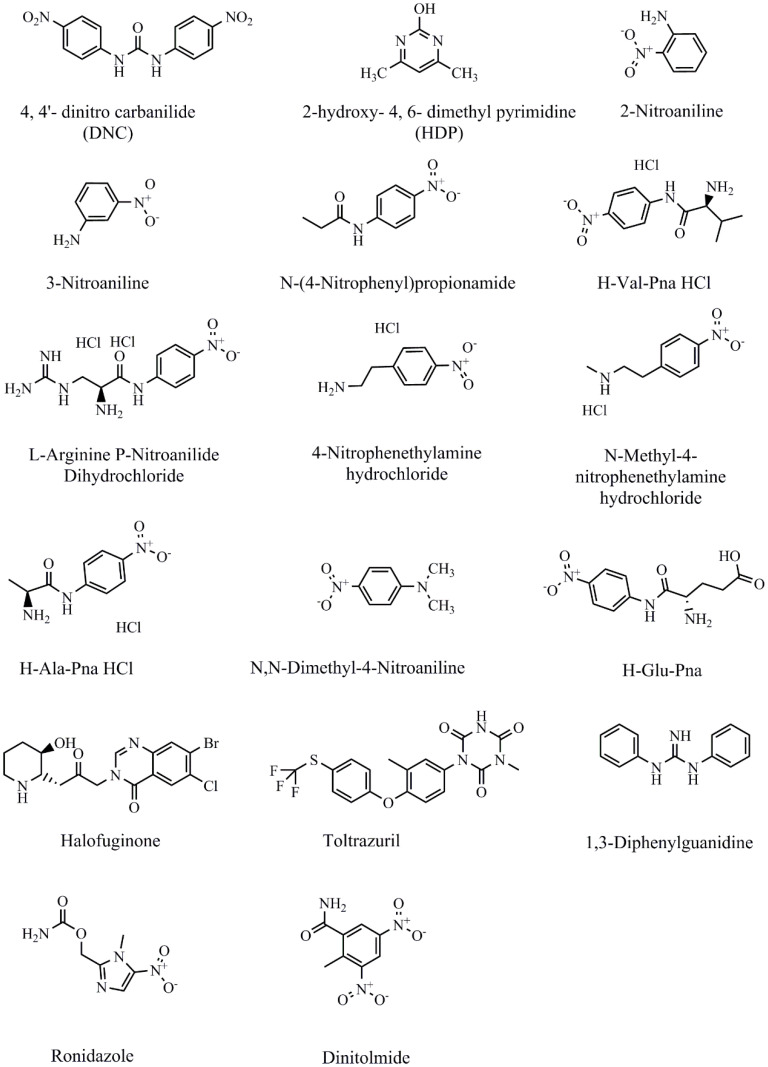
Chemical structures of NIC and other analytes.

NIC, 4-nitroaniline, 2-nitroaniline, 3-nitroaniline, N-(4-nitrophenyl) propionamide, H-Val-pNA HCl, L-argininep-nitroanilide dihydrochloride, 4-nitrophenethylamine hydrochloride, N-methyl-4-nitrophenethylamine hydrochloride, H-Ala-pNA HCl, N, N-dimethyl-4-nitroaniline, H-Glu-pNA, halofuginone, toltrazuril, 1,3-diphenylguanidine, ronidazole, and dinitolmide were obtained from Sigma-Aldrich (St. Louis, MO, USA) (Figure 1). The coating antigen (DNC-4-BSA) and mAb 3B4 were acquired from China Agricultural University (Qianqian Tang May 2018). For other reagents materials were shown in the Supplementary Materials part.

### 2.2. Apparatus

Water was purified using a Milli-Q system from Millipore Inc. (Bedford, MA, USA). A NanoDrop 2000 ultraviolet spectrophotometer was purchased from Thermo Scientific (Waltham, MA, USA). An ultraviolet analyzer was obtained from Tianjin Huike Instrument Equipment Co., Ltd. (Tianjin, China). A time-resolved immunochromatography (TRF) reader was supplied by Beijing EDWK BIOTECH (Beijing, China).

### 2.3. Preparation of Eu-NP-mAb Probes and Eu-NP-Chicken IgY Probes

The EuNPs were developed using a modified method, as previously described [22,23]. Carboxylate-activated EuNP surfaces were conjugated with mAb 3B4 or IgY using a typical N-Hydroxysuccinimide (NHS)/ethyl dimethylamine carbonide (EDC) conjugation method, which maintained 4 °C to ensure the retention of mAb activity [22]. The preparation process of EuNPs and mAb 3B4 conjugates is described as follows: In brief, 20 μL of EuNPs (200 nM) was added to 200 μL 50 mM MES (pH 5.0) containing EDC and sulfo-NHS at 0.1 mM and 0.2 mM. The reaction was reacted at room temperature for 15 min, and centrifuged 15,000× *g* at high speed for 10 min at 4 °C to separate the supernatant. After being washed twice, an ultrasound was performed in 200 μL sodium borate for 2 min. The mAb 3B4 or IgY (5 μL) was then added, and the mixtures were shaken before centrifugation. The residue was suspended in 100 μL of 10 mM phosphate buffer and reacted for 2 h at 25 °C. After centrifugation, the residue was suspended in 2 mL 0.2 mM Tris-HCl containing 0.5% PVP (pH 7.4).

### 2.4. TRFICA Procedure

Initially, 200 μL of DNC standard solution or sample extract, Eu-NP-mAb probes, and Eu-NP-chicken IgY probes were injected into the micropore and mixed for 1 min. Afterward, the mixture was added to the sample pad well, and the liquid migrated slowly toward the absorption pad through capillary action. After 8 min of incubation, the fluorescence intensities of the T and C lines were observed by a TRF reader. In the presence of DNC, the absence of fewer Eu-NP-mAb probes was captured by the T line, weakening the fluorescence intensity on the T line, and the Eu-NP-chicken IgY probes were captured by the C line. The quantitative analysis of the DNC was conducted by recording the fluorescence intensities of the T and C lines. The T/C ratio was used to offset the background and inherent heterogeneity of the strip. This was expected to be inversely proportional to the increasing concentration of DNC in the samples. The standard curve was prepared at nine levels of DNC concentration (0, 0.07, 0.19, 0.56, 1.67, 1.37, 5, and 15 ng mL^−1^), and each concentration was tested in triplicate. A four-parameter logistic equation was performed and calculated using Origin 8.0 (Origin Lab, Northampton, MA, USA).

### 2.5. TRFICA Development and Optimization

**Figure 2:**
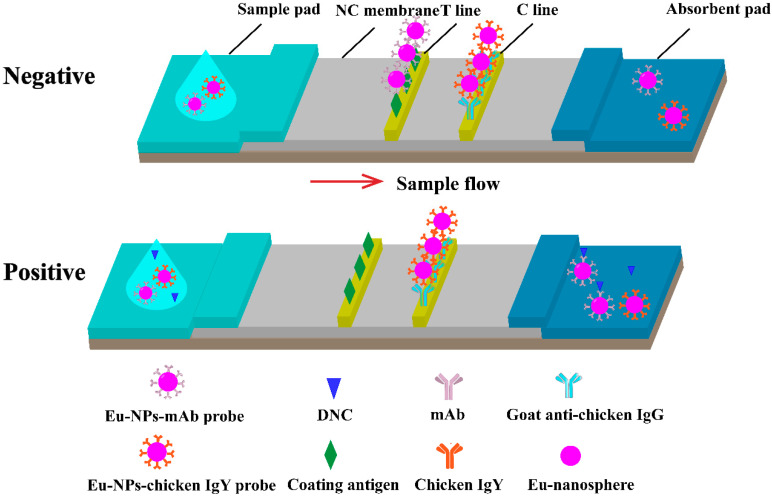
Schematic illustration of TRFICA. The schematic illustrates the working principles of TRFICA. T line is the test line, and the C line is the control line. After dripping the sample, the sample migrated along the NC membrane through capillary action. For a negative sample, the fluorescence intensity of the T line and the C line can be detected by the TRF reader. For a positive sample, only the T line had fluorescence intensity, and the C line had no fluorescence intensity. The surplus probe complexes migrated to the absorption pad.

The immunochromatographic strips used for TRFICA testing were composed of a sample pad, a conjugate pad, an NC membrane coated with capture reagents, an absorbent pad, and a polyvinyl chloride sheet with adhesive tape (Figure 2). The controls included in the strip were DNC-4-BSA conjugate (test ‘T’ line) and goat-anti-chicken IgG (control ‘C’ line), and they were immobilized on the NC membrane and separated by a distance of 3 mm. The coated membrane was dried at 37 °C overnight. The NC membrane, sample pad, and absorbent pad were successively laminated and pasted onto the PVC base plate. The entire assembly was cut into 3–4 mm-wide strips and stored desiccated at room temperature.

The assay was optimized by optimizing single analytical parameters, while the others remained constant. The fluorescence intensity and inhibition rate [(F_0_ − F)/F_0_] × 100% were used to assess the sensitivity of the immunoassays. F and F_0_ were the fluorescence values of DNC at 5 ng mL^−1^ and without DNC, respectively. The tested parameters included the conjugation of Eu-NP-mAb probes, the concentrations of antibody and coating antigen, reconstitution buffers, probe amounts, coating buffers, incubation time, sample dilution buffer, and the immunochromatographic strip type. After optimizing and determining the optimum experimental parameters, a standard curve was obtained by plotting T/C against the DNC concentration. A four-parameter logistic equation was used to calculate the performance parameters.

### 2.6. Curve Fitting and Statistical Analysis

A logistic equation used to fit the TRFICA data are shown in the Supplement information part. The cross-reactivity (CR) with DNC analogizes was determined after the optimized conditions, and the specificity of TRFICA was evaluated. The CR was calculated by the following equation [28]:

CR (%) = (IC_50_ of DNC/IC_50_ of DNC analog) × 100 (1)

### 2.7. Chicken Sample Analysis for TRFICA

Samples of chicken muscle (2 g) were added to 2 mL acetonitrile in 10 mL polypropylene tubes and sonicated for 10 min. The mixture was centrifuged at 8000× *g* for 10 min, and the supernatant was diluted 20-fold with an assay buffer for subsequent TRFICA analysis. Matrix interference was determined by comparing the DNC standard curve prepared in the assay buffer and the chicken extract after a 20-fold dilution with an assay buffer.

Additionally, the accuracy and precision of TRFICA were evaluated and conducted as indicated. DNC-negative chicken homogenate samples were spiked with DNC at 6, 16, and 30 μg kg^−1^. Five replicates were determined at each concentration for both intra-assay and inter-assay determinations.

## 3. Results and Discussion

### 3.1. Development and Optimization of TRFICA

#### 3.1.1. Optimization of the Conjugation of Eu-NP-mAb Probes

**Figure 3:**
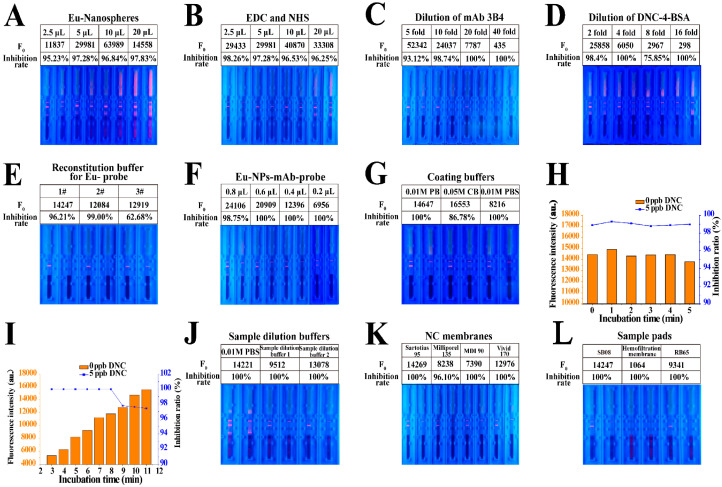
Optimization of TRFICA performance. (**A**) Use of EuNPs. (**B**) Use of EDC and NHS. (**C**) Dilution of mAb 3B4. (**D**) Dilution of DNC-4-BSA. (**E**) Reconstitution buffer for Eu-NP-mAb probes. (**F**) Use of Eu-NP-mAb probes. (**G**) Coating buffer. (**H**) Incubation time for the Eu probe and the DNC. (**I**) Incubation time for immunochromatography. (**J**) Sample dilution buffer. (**K**) NC membranes. (**L**) Sample pads.

An appropriate ratio of the antibody and EuNPs was selected to improve the performance of TRFICA. Thus, the Eu-NP-mAb probes were initially prepared using different mass ratios of the antibody and EuNPs, and the use of the nanospheres at 2.5, 5, 10, and 20 μL was examined. As the EuNPs increased from 2.5 μL to 10 μL, the fluorescent intensity of the T line increased but decreased at 20 μL. Moreover, the chromatogram was incomplete, the fluorescent intensity of the background increased, and the T-line boundary was no longer obvious at 20 μL EuNPs. Although the strongest fluorescent intensity for the T line was obtained at 10 μL EuNPs, the inhibition rate was lower. The inhibition rate was better at 5 μL EuNPs, along with better fluorescent intensity on the T line (Figure 3A).

Typical EDC conjugation methods were used for reagent development, as mild conditions maintained antibody activity. EDC and NHS can directly affect the coupling efficiency of antibodies and EuNPs by activating the carboxyl group on the microspheres, which then allows coupling with mAb amino groups.

We used EDC and NHS at 2.5, 5, 10, and 20 μL, and following optimization, 2.5 μL EDC and NHS were used to activate a 1-mL EuNP solution. This resulted in a better inhibition rate and acceptable fluorescent intensity on the T line (Figure 3B).

**Figure 4:**
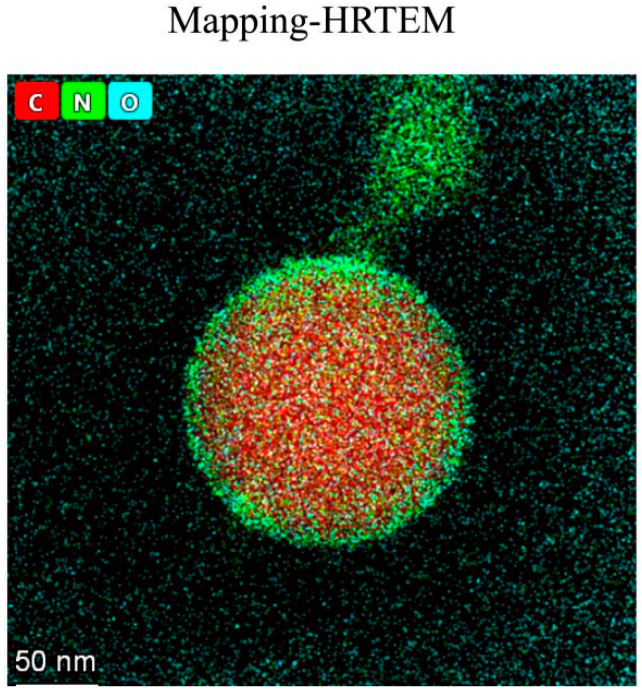
Elemental mapping images of the Eu-NP-mAb. The existence of the N element and the O element observed in the elemental mapping image confirms the successfully modified mAb on the EuNPs.

The Eu-NP-mAb probes were characterized by high-resolution transmission electron microscope studies. After coupling, the EuNP surface modified by the immobilized mAb was characterized by high-resolution transmission electron microscopy. The results validated the distribution of the elements on the surface of EuNPs attached to C, N, O, and other elements (Figure 4). The existence of the N element proved the successful coupling of EuNPs with mAb 3B4.

#### 3.1.2. Optimization of the Concentrations of mAb and Coating Antigen

The specific binding of immunoreagents is the basis of TRFICA, as with other immunoassays. An appropriate antigen–antibody ratio can enhance the binding of specific sites and improve TRFICA performance [29]. Thus, the concentrations of mAb 3B4 and the coating antigen DNC-4-BSA were screened using checkerboard titration. According to the results, a 10-fold dilution of mAb 3B4 was selected due to the acceptable fluorescent intensity inhibition rate (Figure 3C). Moreover, a high dilution of DNC-4-BSA decreased the fluorescent intensity and increased the inhibition rate. Thus, a two-fold dilution of DNC-4-BSA was selected (Figure 3D).

#### 3.1.3. Optimization of the Reconstitution Buffer for Eu-NP-mAb Probes

In this study, we also evaluated three reconstitution buffers for Eu-NP-mAb probes based on the inhibition ratios and F_0_ values (Table S1). According to Figure 3E, the three F_0_ values that were obtained differed little, and the highest inhibition ratio was observed with the reconstitution buffer 2^#^. Therefore, reconstitution buffer 2^#^ was selected.

#### 3.1.4. Optimization of Eu-NP-mAb Probe Usage

The effects of the quantity of Eu-NP-mAb probes present in the TRFICA system were also examined. As the number of Eu-NP-mAb probes decreased, the F_0_ value of the T line also decreased, while the inhibition rate increased only gradually. The F_0_ value was controlled at about 12,000; therefore, 0.4 μL of Eu probe was selected as the optimal amount to be used for the assay (Figure 3F).

#### 3.1.5. Optimization of Coating Buffers

For the optimization of coating buffers, three typical coating buffers were tested in the system: 0.01 M phosphate buffer (PB) at pH 7.4, 0.05 M carbonate buffer (CB) at pH 9.6, and 0.01 M phosphate buffer solution (PBS) at pH 7.4. The F_0_ values decreased dramatically in PBS, whereas both PB and CB resulted in significant increases in F_0_. In particular, the use of PB led to a higher inhibition ratio (Figure 3G). Thus, 0.01 M PB at pH 7.4 was selected as the best coating buffer.

#### 3.1.6. Optimization of Incubation Times

Incubation time can significantly influence the performance of TRFICA. Initially, we examined the incubation times of 0, 1, 2, 3, 4, and 5 min for the DNC standard solution or sample extract, Eu-NP-mAb probes, and Eu-NP-chicken IgY probes. As these times had no significant effect on fluorescence intensity or inhibition ratio, 1 min was selected (Figure 3H). We then compared different incubation times (3, 4, 5, 6, 7, 8, 9, 10, and 11 min) for immunochromatography. We found that longer incubation times enhanced the binding efficiency of the assay (Figure 3I). However, when the incubation time exceeded 9 min, the sensitivity decreased dramatically. Therefore, 8 min was selected as the best incubation time for immunochromatography in this study.

#### 3.1.7. Optimization of Sample Dilution Buffers

Using the appropriate sample dilution buffers for TRFICA resulted in better performance. In this section, three sample dilution buffers were examined: PBS and sample dilution buffers 1 and 2 (Table S2). The use of sample dilution buffer 1 resulted in a decrease in fluorescence intensity, while PBS generated a high background, most likely due to the release of the probes. Thus, sample dilution buffer 2 was selected as the optimal sample dilution buffer, with satisfactory fluorescence intensity and inhibition rate (Figure 3J).

#### 3.1.8. Optimization of Immunochromatographic Strips

The use of the NC membrane affected the TRFICA sensitivity primarily through the membrane pore and the protein binding forces. Four NC membranes, Sartorius 95, Millipore 135, MDI 90, and Vivid 170, as well as their corresponding F_0_ values and inhibition rates, were evaluated. After optimization, the Sartorius 95 membrane was selected as the optimum (Figure 3K). Immunochromatography was also affected by the speed and release effect of the sample pad. Three sample pads, namely SB08, hemofiltration membrane, and RB65 were assessed, and the strongest fluorescence intensity was obtained using sample pad SB08 (Figure 3L).

### 3.2. Sensitivity and Cross-Reactivity of TRFICA

**Figure 5:**
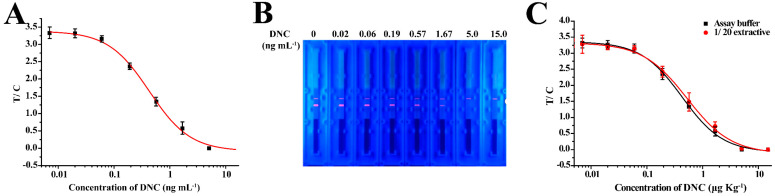
Standard curve of the TRFIA. (**A**) Standard curves of TRFICA for DNC in an assay buffer under optimized conditions. (**B**) Fluorescent image of the TRFICA strip acquired under ultraviolet light for 0, 0.02, 0.06, 0.19, 0.57, 1.67, 5.0, and 15 ng mL^−1^ in an assay buffer (left to right). (**C**) Comparison of the TRFICA curves obtained from the standards prepared in the assay buffer and the 20-fold diluted chicken extracts.

The standard curve of TRFICA in the buffer was established under optimized conditions. To evaluate the sensitivity of the method, the sensitivity of TRFICA was evaluated using the IC_50_, working range, and LOD. The LOD was examined and defined as the IC_10_ from the standard curve. According to curve fitting, the IC_50,_ linear working range, LOD, and cut-off value of the method were 0.43 ng mL^−1^, 0.13–1.37 ng mL^−1^, 0.07 ng mL^−1^, and 5 ng mL^−1^, respectively (Figure 5A,B). These values represent a significantly improved performance relative to previous reports in which the LOD of the colloidal gold-based immunochromatographic test method was 0.86 ng mL^−1^. This indicates that TRFICA performed as an ultrasensitive detector of DNC.

The specificity of TRFICA was determined using cross-reactivity with 15 DNC analogs. As shown in Table 1, the CR was <0.1% and no inhibitions were observed even though the concentration of the standards was 1000 ng mL^−1^. The results of the CRs were highly similar to those from the fluorescence polarization immunoassay (FPIA) using mAb 3B4 [30]. These results indicate that the developed TRFICA was specific to DNC detection.

### 3.3. Chicken Sample Analysis for TRFICA

The chicken muscle was examined for the applicability of the developed TRFICA. In TRFICA, the complexity of the chicken muscle was directly related to the sensitivity and accuracy of the immunoassay. In this study, organic solvents were chosen as the extraction solvent for DNC from muscle tissues. We initially examined seven extraction solvents (Table S3). However, with the exception of acetonitrile, all the extraction solvents resulted in significant decreases in F_0_ values. Therefore, acetonitrile was selected as the extraction buffer for further investigation. The standard curves of DNC in chicken muscle extracts using acetonitrile after 20-fold dilutions and when included in the assay buffer sufficiently reduced the matrix effects (Figure 5C). Under optimized conditions, this allowed the detection of DNC with a LOD and working range of 1.4 and 2.96–37.06 μg kg^−1^, respectively. The total analysis time, including the sample pre-treatment, was less than 30 min, which has not been achieved in other immunoassays, such as ELISA [15,16,17], FPIA [30], and the colloidal gold-based immunochromatographic test method [18]. Thus, due to its efficiency and easy operation, TRFICA was better suited for the analysis of DNC.

To further evaluate the utility of TRFICA, we determined the DNC recoveries from the chicken matrix. All samples were fortified with DNC at three levels (6, 16, and 30 μg kg^−1^), and the average recoveries ranged from 77.3% to 92.7%, with a coefficient of variation (CV) < 7.0% (Table 2). This perfectly met the requirements for DNC residue detection. Therefore, the developed method is suitable for the determination of DNC residues in chicken muscle.

## 4. Conclusions

In summary, a TRFICA was developed for the simple, sensitive, and quantitative detection of DNC in chicken samples. The optimization of the nanosphere–probe conjugation, concentrations of antibody and coating antigen, reconstitution buffers, probe amounts, coating buffers, incubation times, sample dilution buffers, and immunochromatographic strip types was clearly demonstrated in this study. The performance of TRFICA was significantly improved under optimum conditions. Notably, the reliability and robustness of the assay were successfully demonstrated for the analysis of DNC in chicken muscle matrices without complicated pre-treatment processes. Furthermore, the total analysis time, including sample pre-treatment, was less than 30 min, which had not yet been achieved in other immunoassays for DNC residues.

## Figures and Tables

**Table 1:** IC_50_ values and cross-reactivity of DNC and 15 structurally related analogs for TRFICA.

Analogues	IC_50_ (ng mL^−1^)	CR (%)
DNC	0.43	100
2-Nitroaniline	>1000	<0.1
3-Nitroaniline	>1000	<0.1
N-(4-Nitrophenyl) propionamide	>1000	<0.1
H-Val-pNA HCl	>1000	<0.1
L-Arginine P-Nitroanilide Dihydrochloride	>1000	<0.1
4-Nitrophenethylamine hydrochloride	>1000	<0.01
N-Methyl-4-nitrophenethylamine hydrochloride	>1000	<0.1
H-Ala-pNA HCl	>1000	<0.1
N, N-Dimethyl-4-Nitroaniline	>1000	<0.1
H-Glu-pNA	>1000	<0.1
Halofuginone	>1000	<0.1
Toltrazuril	>1000	<0.1
1,3-Diphenylguanidine	>1000	<0.1
Ronidazole	>1000	<0.1
Dinitolmide	>1000	<0.1

**Table 2:** Recovery studies from chicken muscle matrices using TRFICA.

Sample	Spiked (ug kg^−1^)	Intra-Assay (*n* = 5)		Inter-Assay (*n* = 5)	
		Recovery (%)	CV (%)	Recovery (%)	CV (%)
Chicken	6.0	77.3	2.5	80.3	4.0
	16.0	81.9	2.4	88.9	3.8
	30.0	87.6	7.0	92.7	5.4

## Data Availability

Not applicable.

## Supplementary Materials

The following supporting information can be downloaded at: https://www.mdpi.com/article/10.3390/bios13050518/s1, Table S1: Three reconstitution buffers for the Eu3+- labeled antibody; Table S2: Sample dilution buffers used in TRFICA; Table S3: Seven extraction solvents used in TRFICA.
