# Supplementary material for: A Europium Nanosphere-Based Time-Resolved Fluorescent Immunochromatographic Assay for the Rapid Screening of 4,4′-Dinitrocarbanilide: Aiming at Improving Strip Method Performance
Source: Biosensors (Basel). 2023 May 4;13(5):518. doi: 10.3390/bios13050518 (PMC10216060; doi:10.3390/bios13050518)
Supplement: Supplementary file 1 [file biosensors-13-00518-s001.zip › biosensors-2216095-supplementary.pdf]

biosensors

A Europium Nanosphere-based Time-resolved Fluorescent Immunochromatographic Assay for the Rapid Screening of 4, 4′-Dinitrocarbanilide in Chicken Muscle: Aiming at Improving Strip Method Performance

Ming Zou [1, a], Yongkang Yin [1, a], Liuchuan Guo [1, a], Qidi Zhang [1, a], Jinyan Li [1, a], Hong Zhang [1, a], Qian Song [1, a], Zhaojie Li [1, a], Li Wang [1, b], Xiang Ao [c], Xiao Liang [a, c*]

[a] College of Veterinary Medicine, Qingdao Agricultural University, No. 700 Changcheng Road, Qingdao 266109, People's Republic of China

[b] College of Food Science and Engineering, Qingdao Agricultural University, No. 700 Changcheng Road, Qingdao 266109, People's Republic of China

[c] Basic Medical College, Qingdao University, No. 308 Ningxia Road, Qingdao 266071, People's Republic of China

[*] Author to whom correspondence should be addressed:

Xiao Liang Tel: +8613964867357; E-mail: liangxiao4000@163.com

Table S1. Three reconstitution buffers for the Eu^{3+}- labeled antibody.

Reconstitution buffer	Composition	Usage
1#	BSA	1 g
	Sucrose	3 g
	Prolin 300	2 μL
	Borate buffer (0.02 M)	100 mL
2#	BSA	1 g
	PEG 20000	0.05 g
	Prolin 300	2 μL
	0.01 M PBS (pH 7.4)	100 mL
3#	Tris	0.605 g
	BSA	5 g
	Sucrose	10 g
	Trehalose	10 g
	Prolin 300	10 μL
	H_2O	500 mL

Table S2. Sample dilution buffers used in TRFICA.

Sample dilution buffer	Composition	Usage
0.01 M PBS	Na_2HPO_4•$12H_2O$	0.290 g
	NaH_2PO_4•$2H_2O$	0.024 g
	NaCl	0.800 g
	KCl	0.020 g
	H_2O	1,000 mL
Sample dilution buffer 1	Tween-20	50 μL
	0.2 mM Tris-HCl	10 mL
Sample dilution buffer 2	PVP	0.050 g
	0.2 mM Tris-HCl	10 mL

Table S3. Seven extraction solvents used in TRFICA.

Extraction solvents	V: V
Acetonitrile	1
Methanol	1
Acetonitrile: Trichloroacetic acid	1: 3
Acetonitrile: Trichloroacetic acid	1:1
Acetonitrile: Trichloroacetic acid	3:1
Ethyl acetate	1
Dichloromethane	1
